# Editorial: Sudden Cardiac Death and Channelopathies

**DOI:** 10.3389/fcvm.2020.605834

**Published:** 2020-11-25

**Authors:** Giannis Baltogiannis, Giulio Conte, Juan Sieira, Gaetano M. De Ferrari, Pedro Brugada

**Affiliations:** ^1^Heart Rhythm Management Center, Vrije University Brussel, Brussels, Belgium; ^2^Heart Rhythm Management Center, Brussels, Belgium; ^3^Division of Cardiology, Fondazione Cardiocentro Ticino, Lugano, Switzerland; ^4^Department of Cardiology, University of Turin, Turin, Italy

**Keywords:** sudden cardiac death (SCD), channelopathies, arrhythmias, genetics, risk stratification

Sudden cardiac death has serious consequences for the patient's relatives and for society. Each year in Europe and the USA, around 350,000 people, or around 0.1% of the general population, have an out-of-hospital cardiac arrest, of which only a small percentage survive with no sequelae.

The etiology of sudden death has been extensively studied at a population level. Ischemic heart disease occupies a prominent position and is responsible for up to 70% of these deaths; other structural heart diseases make up 10%, and primary arrhythmias cause a further 10%.

In young patients (less than 35 years of age), in whom the incidence of sudden death is 100 times lower than in the general population, arrhythmic etiology, mainly from channelopathies, in the absence of structural heart disease is much more common and is the predominant cause of sudden death in patients aged between 14 and 25 years. In older patients channelopathies such as Brugada syndrome are more likely to occur in the fourth decade of life.

The channelopathies responsible for sudden cardiac death are: Long QT syndrome, Short QT syndrome, Brugada syndrome, Catecholaminergic Polymorphic Ventricular Tachycardia syndrome, and Early repolarization Syndrome.

Early identification and risk stratification is of major importance in patients with a channelopathy who remain asymptomatic, for several reasons. First, sudden death may be the first manifestation of the disease, with no previous warning symptoms. In addition, it should be considered that when we diagnose a patient with a hereditary disease, we also diagnose their family. Identification of an individual with this condition must be accompanied by meticulous familial screening. It is true that in these diseases the risk of sudden death decreases with age, but even a diagnosis in an elderly patient is relevant, as it allows identification of the disease in relatives and their appropriate work-up.

The scope of the Research Topic is to collect state-of-the art papers on what we know so far for all types of Long QT syndromes, Brugada Syndrome, Early repolarization Syndrome, Catecholaminergic Polymorphic Ventricular Tachycardia, and Short QT syndrome. Furthermore, our aim is to provide further insight on the current research areas, and which should be the area's future perspectives.

Ezeani explored one of the numerous mechanisms with which the arrhythmias present. Identifying the sources that control Ca^2+^ is novel in understanding arrhythmogenesis. The TRP channels mediate Ca^2+^ flux and voltage changes across membranes. Regulation of Ca^2+^-handling by the TRP channels indicate that they can potentially boost Ca^2+^ cycling disorders. The plasma membrane sensory and metabotropic TRPM4 subgroup is a drug candidate for Brugada syndrome and familial heart blockers.

Novelli et al. dealt with *Pleiotropic Phenotypes Associated with PKP2 Variants*. Their review on plakophilin-2 (PKP2) coded by the gene Pkp2, whose pathogenic role has recently been recognized in different inherited cardiac arrhythmias syndromes, ranging from Arrhythmogenic Cardiomyopathy (ACM or ARVC), Brugada Syndrome (BrS), idiopathic ventricular fibrillation, hypertrophic cardiomyopathy (HCM), and dilated cardiomyopathy (DCM). The expansion and increased availability of genetic testing has challenged the concept of “one gene-one disease” and has shown that different phenotypes can be caused by variants on one same gene. The interpretation of these findings in light of human variation data is complex and casts some warning on the clinical application of this information. The evidence of the pleiotropy of a gene suggested by genetic variants and their functional effect *in vitro* has the important value of discovering different protein functions and suggest arrhythmia mechanisms.

Badone et al. contributed to the field by reviewing the Functional Effects of Calmoduline Mutations and Their Relationship with Clinical Phenotypes. Based on the information reviewed above, mechanism-guided therapeutic approaches to calmodulinopathies should ideally address the interaction of mutant CaM with its targets. Particularly in the case of LQTS-type mutations, this approach is justified by the role of the high target affinity of mutant CaMs in causing negative dominance of the mutation. Tools for this purpose are not available yet, but possibilities exist and are currently explored.

Kotta et al. present in a detailed CaM's sequence, structure and function, the genetic spectrum of CaM mutations and the associated phenotypes, as well as available therapies. They also provide an overview of the underlying disease mechanisms of calmodulinopathy (reviewed in detail in the accompanying article by Badone et al.) and present the thus far used *in vitro* methods for deciphering these disease mechanisms.

Casado Arroyo et al. provided an overview for the current evidence supporting different theories explaining Early Repolarization Syndrome. Along with future developments in the field directed toward individualized treatment, strategies are also examined. Bourier et al. add significantly to the field of ER syndrome, suggesting a risk stratification approach and therapeutic management.

Baltogiannis et al. presented Catecholaminergic Polymorphic Ventricular Tachycardia (CPVT) in this collection, as arrhythmogenesis, therapeutic Management, and Future Perspectives are still challenging. Dutsi et al. provided, in detail, the therapeutic impact of Cardiac Sympathetic Denervation in Cardiac Channelopathies. clinical data from well-conducted multicenter registries largely confirmed the preclinical findings, showing that LCSD is an effective treatment for drug-refractory ventricular arrhythmias in both LQTS and CPVT, and that LCSD is now recommended in recent guidelines. Not surprisingly, considering the mechanism of action, the efficacy and potential indication of LCSD in channelopathies goes far beyond secondary prevention, potentially including many still asymptomatic patients with high-risk features for SCD, despite optimized medical therapy.

Campuzano et al. offered a mini review in Recent Advances in Short QT Syndrome. Nearly 20 years ago, SQTS was reported as a familial arrhythmogenic entity. Nowadays, a low number of families have been reported but with a high lethality. This lack reported families, impedes the establishment of a conclusive risk stratification scale, particularly in asymptomatic cases carrying a genetic alteration. New development of hiPSC-CMs from patients may allow unraveling pathophysiological mechanism, helping to understand or treat the disease. Patients suffering of SQTS are at high risk of syncope and SCD. Implantation of an ICD remains the most effective preventive measure after aborted SCD and malignant ventricular arrhythmia, although pharmacological therapies may be used in certain cases, especially in children. Currently, regardless of advances being made in genetics, almost 70–80% of families remain without a genetic cause identified after a comprehensive analysis. Genotype-phenotype analysis are necessary to improve current guidelines in early identification as well as prevention in families suffering from SQTS. On the other hand, Wilders and Verkerk dealt with Long QT Syndrome and Sinus Bradycardia. Sinus bradycardia has been reported in relation to a large number of LQTS mutations. The occurrence of both QT prolongation and sinus bradycardia on a family basis is almost completely limited to LQT3 and Ankyrin-B syndrome (“LQT4”). However, the mechanisms of the associated ventricular arrhythmias and sudden death are largely different. Cardiac events, including nocturnal sudden death, are provoked by the bradycardia and associated excessive QT prolongation in case of LQT3, whereas disturbed calcium homeostasis leads to dysfunction of the SAN cells in case of the Ankyrin-B syndrome, with sudden death occurring after physical exertion and emotional stress.

Moreau and Chahine have added significant value on the topic by introducing a New Cardiac Channelopathy Associated with Na_v_1.5 Gating Pores. The potential cardiac cellular effects of gating pores and their blockers are also presented here. The increasing knowledge regarding gating pores and their pathologic implication, potentially highlights novel biophysical defects and consequently novel channelopathies. In the current dynamic toward more precise and personalized medicine, this growing knowledge could in the future orientate clinicians in their day to day practice in the management of cardiac channelopathies. This could also help to develop specifically targeted novel medication to accurately and precisely block gating pores, finally benefitting patients.

Verkerk et al. analyzed all possible disease modifiers of Inherited SCN5A Channelopathy. Genetic modifiers, (common) co-morbidities, environmental influences, and life style factors including diet and exercise may modify disease expressivity and severity, and as such significantly modulate the risk for arrhythmia occurrence and survival in *SCN5A* channelopathy. Importantly, the impact of modulatory factors may differ between distinct mutations but may also vary with age and gender. Hence, the clinical management of patients with *SCN5A* mutations should include careful and continuous assessment of co-existing diseases and other modulatory factors, in addition to rigorous treatment of relevant co-morbidities. Identification of disease modifiers will be an essential step in further research related to *SCN5A* channelopathies and may help to design better risk stratification algorithms and to improve development of novel diagnostic and therapeutic strategies. Tse et al. provided electrocardiographic evidence that higher levels of dispersion in conduction and repolarization are found in type 1 than non-type 1 BrS patients. This may potentially explain the higher incidence of ventricular arrhythmias in the former group. Indices reflecting cumulative conduction and repolarization abnormalities may provide additional value for risk stratification.

Finally, Cheniti et al. dealt with Mapping and Ablation of Idiopathic Ventricular Fibrillation. Insights in pathophysiology of idiopathic VF are presented. Idiopathic VF is diagnosed in around one third of survivors of unexplained SCD aged under 35 years. Genetic testing allows identification of a likely causative mutation in around one quarter of unexplained sudden deaths in children and young adults. Ablation of the PVCs that trigger VF in this setting is associated with high rates of acute success and long-term freedom from VF recurrence. Importantly, almost two thirds of patients have subtle structural abnormalities identified by high density electrogram mapping which are missed by current imaging tools. This localized substrate, which co-locates with regions of VF drivers, provides an explanation for so called unexplained SCD and represents a novel potential target for ablation.

## Author Contributions

GB has written the editorial. GC, JS, GD, and PB have edited the editorial. All authors contributed to the article and approved the submitted version.

## Conflict of Interest

The authors declare that the research was conducted in the absence of any commercial or financial relationships that could be construed as a potential conflict of interest.

